# Tackling the persistent burden of tuberculosis among people living with HIV

**DOI:** 10.7448/IAS.19.1.21002

**Published:** 2016-03-24

**Authors:** Haileyesus Getahun, Nathan Ford

**Affiliations:** 1 Global Tuberculosis Programme, World Health Organization (WHO), Geneva, Switzerland; 2 Department of HIV and Global Hepatitis Programme, WHO, Geneva, Switzerland

The past decade has delivered major advances to reduce the burden of tuberculosis (TB) among HIV-positive individuals. New molecular technologies have improved the availability and accuracy of TB diagnosis; there are now 16 million people living with HIV on antiretroviral therapy (ART), which reduces both TB-associated illness and risk of infection; uptake of isoniazid preventive therapy continues to increase; outcomes of TB treatment are improving; new drugs and regimens with the potential to shorten treatment against drug-resistant TB have been developed; and there have been advances in service delivery approaches to tackle both diseases, notably integration of TB and HIV services, community-based service delivery and the involvement of community health workers and civil society organizations in providing patient support, including counselling, testing, health education and advocacy.

The availability of new drugs, diagnostics and service delivery approaches has translated into a reduction in the number of people dying from HIV-associated TB globally, with a fall in TB mortality by 32% between 2004 and 2014 [1]. Yet, TB persists as the most important co-morbidity among people living with HIV. Generally, people living with HIV are 26 times more likely to develop TB than those who are HIV negative; 12% of the 9.6 million new TB cases reported globally in 2014 were HIV positive; while all regions are affected, this figure rises to above 70% in some high-burden countries in southern Africa [2], and a high burden of co-infection is found in populations where risks of acquisition are shared, notably prisoners, migrant populations, and mine workers. TB remains a leading cause of HIV-associated hospitalization and of death among adults and children living with HIV worldwide [3], and accounted for a third of the estimated 1.2 million HIV-related deaths globally in 2014, the majority of these deaths (190,000) occurring among men [2].

In order to further reduce the burden of TB among people living with HIV, four priorities stand out.

First, the awareness of disease status among people living with HIV needs to be improved, through better coverage of routine HIV testing and TB screening. The World Health Organization (WHO) recommends that routine HIV testing should be offered to all patients known or suspected to have TB, including partners of HIV-positive TB patients. Yet, in 2014, only 3.2 million (51%) of notified TB patients had an HIV test, with this figure rising to 79% in the African region. HIV testing expansion efforts need to be combined with TB screening activities. People living with HIV should be systematically screened for TB. The number of HIV-positive people screened for TB, and the number of countries reporting data, are both increasing, from 5.5 million in 2013 (64 reporting countries) to 7 million in 2014 (78 reporting countries), but further improvements are needed. This is particularly important given the persistent problem of late presentation to care, with CD4 cell count at presentation to care below 200 cells/mm^3^ in countries with a high burden of TB and HIV [4]. Late presentation to HIV care also points to the need to improve access to earlier testing for HIV.

Second, there are opportunities to improve the diagnosis of TB among people living with HIV. HIV changes the presentation of TB that is harder to detect with conventional methods – reduced pulmonary cavity formation and sputum bacillary load and a more frequent involvement of the lower lobes and organs other than the lungs [5]. Diagnostic algorithms to speed up the diagnosis and initiation of treatment should be routinely used. The initial use of molecular-based TB diagnosis as part of HIV services should be routinized. Tests based on the detection of mycobacterial lipoarabinomannan (LAM) antigen in urine have emerged as potential point-of-care tests for TB and have showed promising results in HIV-positive individuals with very advanced disease. WHO recommends lateral flow LAM to assist and expedite the diagnosis of TB in two specific population groups: in HIV-positive, adult in-patients with signs and symptoms of TB (pulmonary and/or extrapulmonary) and a CD4 cell count ≤100 cells/mm^3^, and people living with HIV who are seriously ill and in respiratory distress (regardless of CD4 count or if the CD4 count is unknown) [6]. The continuous vigilance of health workers in suspecting TB among people living with HIV is extremely important to shorten the long delay in health service diagnosis and associated higher risk of death [7].

Third, access to isoniazid preventive therapy needs to improve. A recent study from West Africa confirmed the benefit of isoniazid preventive therapy which, when given with ART to people with high CD4 cell counts, reduced the risk of severe HIV-related illness by 44% and the risk of death by 35% [8]. While coverage of co-trimoxazole preventive therapy is high, at around 87% of all notified HIV-positive TB patients, coverage of isoniazid preventive therapy in newly enrolled in HIV care is below 41% among the high TB/HIV burden countries that reported data in 2014, and was much lower in some countries, for example just 5% in Swaziland. Most importantly, the use of shorter rifamycin-containing regimens in high TB and HIV burden settings should be explored and encouraged.

The fourth priority is to improve the prompt access to ART for those TB patients who are identified to be HIV positive. ART is strongly associated with a reduction in TB incidence [9], and improved outcomes among HIV-positive individuals who have active TB and advanced immune deterioration [10]. Since 2010, WHO has recommended starting ART as soon as possible (within two to eight weeks) among TB patients who are found to be HIV positive [11], and while ART coverage is increasing among TB patients, still around a quarter (23%) of identified HIV-positive TB patients worldwide were not receiving ART in 2014 [2].

Recent recommendations issued by WHO in 2015 provide opportunities to further improve coverage of testing, treatment and prevention.

For HIV testing, since 2013, WHO has recommended a broader approach to testing, including community- and home-based testing approaches. Self-testing is an additional approach that is gaining momentum as a way to reach people earlier in their HIV infection, including people who would not normally be reached through traditional clinic-based approaches. WHO is in the process of consolidating these different approaches and will issue updated testing guidance in mid-2016. For TB testing, WHO is working with partners to encourage the use of Xpert/MTB RIF as the initial diagnostic test in order maximize the detection of TB cases among HIV-positive people, including in peripheral facilities [12].

For prevention, evidence supports extending the duration of both co-trimoxazole and isoniazid preventive therapy for people living with HIV at risk of developing TB, and this is reflected in the latest WHO guidance [13]. To support delivery of preventive therapy, a fixed-dose, single tablet coformulation of co-trimoxazole and isoniazid is under development and is anticipated to become available in late 2016 [14].

For ART, new guidance issued in late 2015 now recommends treating of all HIV-positive individuals irrespective of their disease status, and this is anticipated to further reduce the risk of TB, incidence, illness and death among HIV-positive patients [15].

This last recommendation in particular has created new momentum around the need to provide timely access to testing, treatment and care for all people living with HIV irrespective of disease status. The End TB Global Strategy and the 90-90-90 Fast Track treatment targets for HIV provide the political framework for getting there. If achieved, these targets will make a significant impact in further reducing TB incidence, illness and deaths among people living with HIV.
